# Primary Myelofibrosis Presenting as Extramedullary Hematopoiesis in a Transplanted Liver Graft: Case Report and Review of the Literature

**DOI:** 10.1155/2016/9515404

**Published:** 2016-01-17

**Authors:** Ghulam Rehman Mohyuddin, Abdulraheem Yacoub

**Affiliations:** ^1^Department of Internal Medicine, Kansas University Medical Center, 3901 Rainbow Boulevard, Kansas City, KS 66103, USA; ^2^Department of Hematology-Oncology, Kansas University Medical Center, 3901 Rainbow Boulevard, Kansas City, KS 66103, USA

## Abstract

Primary myelofibrosis (PMF) commonly results in extramedullary hematopoiesis (EMH) in the spleen and liver as well as a variety of other organs. We present a first report of a unique presentation of PMF in a liver transplant recipient patient as EMH in the transplanted liver graft. A 76-year-old man with history of cryptogenic cirrhosis received cadaveric liver transplantation in 1996. He maintained a normal graft function and stable hematologic parameters until 2013 when he presented with anemia and progressive fatigue. Extensive work-up did not identify the etiology of the recent decline in his hemoglobin; thus a liver biopsy was done which showed findings of EMH within the sinusoids with increased megakaryocytes, some with atypical morphology. A BM biopsy revealed a hypercellular marrow, moderately increased reticulin fibrosis, and features consistent with primary myelofibrosis. Abdominal imaging showed a normal-size spleen and did not identify any sites of EMH outside of the liver. The diagnosis of myelofibrosis was thus made, and this case demonstrated predominant tropism to a transplanted liver graft with absence of EMH elsewhere. We would thus like to emphasize that findings of EMH in subjects with no preexisting hematologic neoplasm should warrant close follow-up and assessment.

## 1. Introduction

Classified as a BCR-ABL negative myeloproliferative neoplasm [1], myelofibrosis is a clonal cell malignancy characterized by progressive bone marrow fibrosis and ineffective erythropoiesis [2].

Extramedullary hematopoiesis is a well-recognized phenomenon of this disease process. Although typically seen in sites of fetal hematopoiesis, it can be found in any organ and present in a myriad of different ways [3].

The pathophysiology of extramedullary hematopoiesis is thought to be associated with the constitutive mobilization of CD34+ cells into the peripheral blood. This dysregulation of hematopoietic stem cell (HSC) trafficking likely ultimately leads to the seeding of extramedullary sites [4]. We present a first report of a unique presentation of PMF in a liver transplant recipient patient as EMH in a transplanted liver graft.

## 2. Case Description

A 76-year-old gentleman presented to our clinic, with complaints of fatigue and shortness of breath on exertion. He had a history of cryptogenic cirrhosis for which he had underwent cadaveric liver transplantation seventeen years ago. Other comorbidities included diabetes mellitus, hypertension, coronary artery disease, and chronic kidney disease, which was thought to be due to chronic use of calcineuric inhibitors.

Other pertinent symptoms included easy bruising. Patient denied chest pain, leg swelling, frequent infections, fever, chills, appetite change, or unexpected weight change.

Physical examination was pertinent for tachycardia. No hepatosplenomegaly or lymphadenopathy was appreciated. Cardiovascular and respiratory examination were unremarkable.

Since 2009, the patient was displaying macrocytic anemia and thrombocytopenia. This had been worsening progressively from a baseline hemoglobin of 12 g/dL in 2009 to 8-9 g/dL in 2013. Since 2010, a thrombocytopenia of 100–150 K/UL had also been observed. The white blood cell count was within normal limits initially and then increased progressively to 59.5 K/UL with left shifted granulopoieses. Rare nucleated red blood cells were observed on the peripheral smear. Folate and Vitamin B-12 laboratory results were normal. There was mild thrombocytopenia with normal morphology. Serum LDH was increased. Iron studies were not suggestive of iron deficiency. An endoscopy and colonoscopy had failed to reveal an active source of bleeding. The patient had received Vitamin B-12, Folate supplementation, and Epoetin-Alfa injections for his anemia.

As a definitive etiology had not been established, graft dysfunction and antirejection therapy were implicated as potential causes of this hematological process. Although liver function tests remained within normal limits, a liver biopsy was performed to determine the status of the liver and to further guide antirejection therapy.

**Figure 1:**
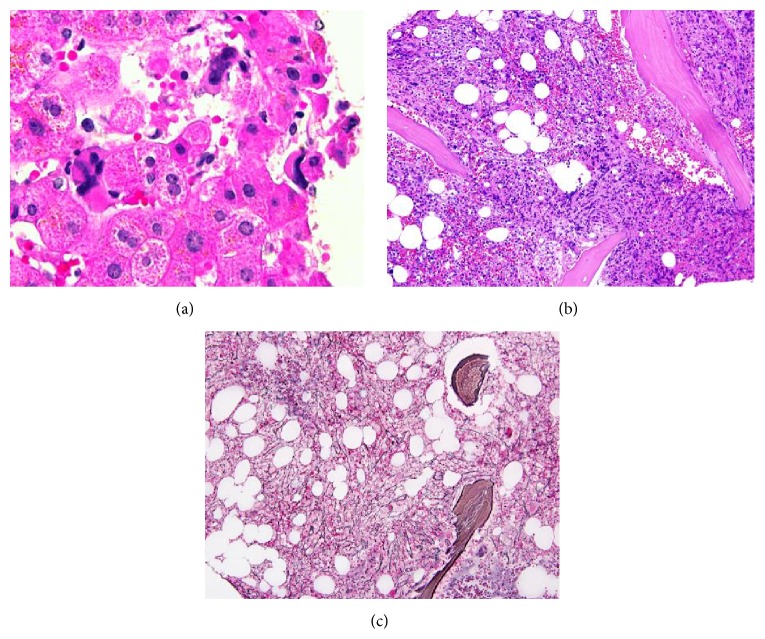
Morphologic findings in liver and bone marrow biopsy. (a) H&E stain of liver, (b) H&E stain of bone marrow biopsy, and (c) reticulin stain of bone marrow biopsy.

**Figure 2:**
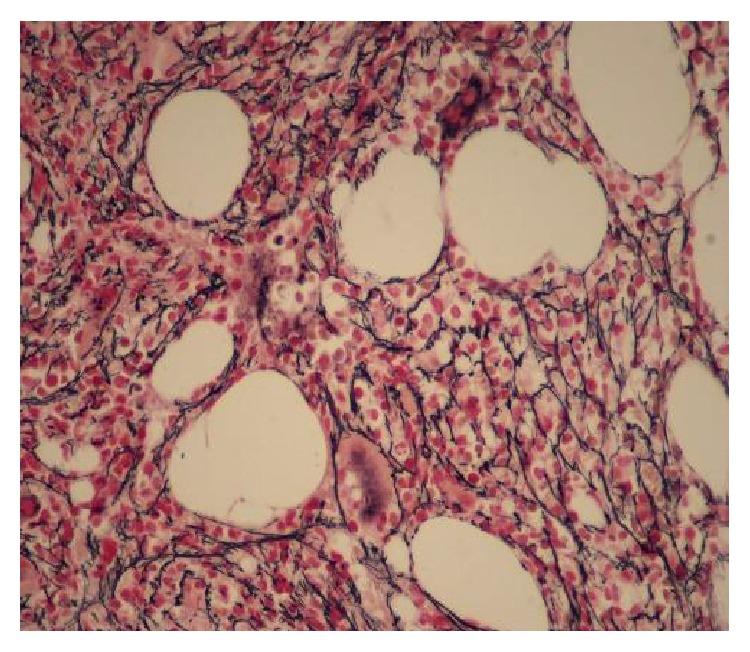
Reticulin stain of the bone marrow biopsy showing increased fibrosis.

**Figure 3:**
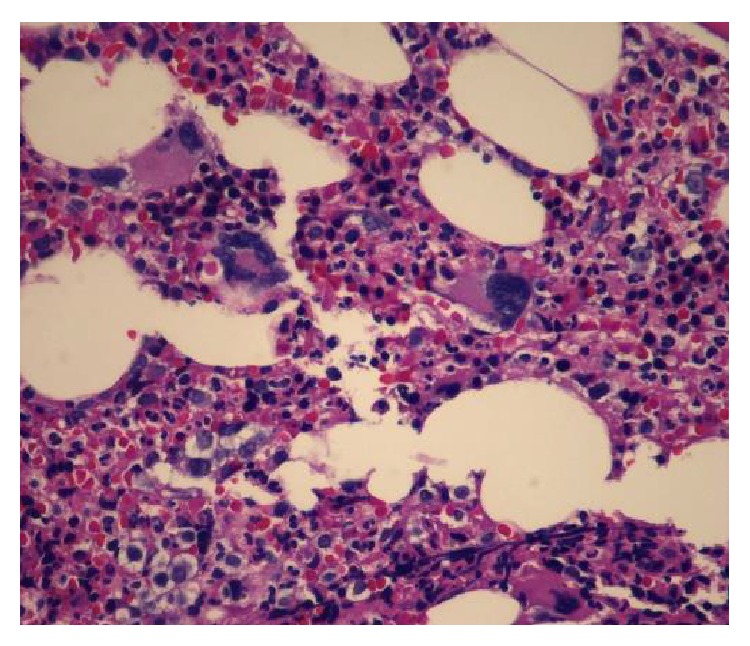
H&E stain of the bone marrow biopsy showing atypical megakaryocytes.

The liver biopsy showed findings of extramedullary hematopoiesis within the sinusoids with increased atypical megakaryocytes. The liver parenchyma was unremarkable with no evidence of rejection or increased fibrosis (Figure 1). Additional work-up included a bone marrow biopsy that revealed a hypercellular marrow (60 percent), polymorphous trilineage hematopoiesis, moderate to severe reticulin fibrosis (grade 2/3), and 1% blasts (Figure 2). The number of megakaryocytes was not markedly abnormal but showed clustering on a subsequent bone marrow biopsy (Figure 3). Cytogenetic studies on the marrow aspirate showed abnormal karyotype: 47, XY, trisomy 8, and add (9) (q34). Polymerase chain reaction (PCR) analysis on the blood for JAK2 mutation was positive for V617F. Abdominal imaging showed a normal-size spleen and did not identify any sites of EMH outside of the liver. The diagnosis of intermediate-2 risk PMF was made by meeting all major criteria and 3 minor criteria.

The patient was classified as intermediate-2 risk based on the Dynamic International Prognostic Scoring System (DIPSS) given his age, anemia, and the presence of constitutional symptoms. He started therapy with Ruxolitinib, a JAK inhibitor, achieving clinical benefit and improvement in symptoms and appetite, and was able to tolerate therapy for 14 months when he progressed. He passed away 25 months after the diagnosis with progressive bone marrow failure.

## 3. Discussion

Our patient is the first presentation of myelofibrosis in a liver transplant recipient patient as extramedullary hematopoiesis (EMH) in a transplanted liver graft, even prior to systemic manifestations of a myeloproliferative neoplasm. In addition, this case demonstrated predominant tropism to a transplanted liver graft. The megakaryocytic atypia and clustering were even more pronounced in the liver compared to bone marrow in our patient, which indicates that the unique microenvironment and immune dysregulation may have facilitated the expansion of this malignant clone. The next question thus follows: is there a unique microenvironment within the transplanted organ that facilitates expansion of malignant clones, as seen in our patient? This definitely merits further exploration.

Interestingly, EMH has been reported in patients who have had liver grafts despite having no bone marrow pathology. The authors postulated various theories to explain this, including local factors within the graft. This was usually seen shortly after transplantation, and the stress of the operative procedure and the ensuing blood loss was seen as a strong contributing factor. However, the authors observed no corelation of preoperative or postoperative hemoglobin levels, graft function, kidney function, and immunosuppressive medication with the presence or absence of erythropoiesis in the grafts. This led them to conclude that intrahepatic erythropoiesis could occur transiently in human liver allografts [5].

The only other report of myelofibrosis presenting as extramedullary hematopoiesis in a transplanted organ that we found in the English literature was that of a patient who was doing well after renal transplant until he presented with new onset proteinuria and rising creatinine. He was ultimately found to have myelofibrosis. On pathology, the appearance was similar to that of acute rejection, with the immature myeloid cells, megakaryocytes, and nucleated red blood cells, easily mistakable as lymphocytes [6]. Given that the management for a transplant rejection would be very different, it is important to entertain the possibility of extramedullary hematopoiesis in such patients, especially when confronted with other signs of bone marrow failure.

## 4. Conclusion

We conclude by emphasizing that findings of extramedullary hematopoiesis in EMH in subjects with no preexisting hematological neoplasms warrant close follow-up and assessment.
